# Testing the Distraction Hypothesis: Do extrafloral nectaries reduce ant‐pollinator conflict?

**DOI:** 10.1111/1365-2745.13135

**Published:** 2019-03-06

**Authors:** Nora Villamil, Karina Boege, Graham N. Stone

**Affiliations:** ^1^ Ashworth Laboratories, Institute of Evolutionary Biology University of Edinburgh Edinburgh UK; ^2^ Instituto de Ecología, Departamento de Ecología Evolutiva Universidad Nacional Autónoma de México Ciudad de México Mexico

**Keywords:** ant‐pollinator conflict, Distraction Hypothesis, extrafloral nectaries, fitness, myrmecophile, *Turnera velutina*

## Abstract

1. Ant guards protect plants from herbivores, but can also hinder pollination by damaging reproductive structures and/or repelling pollinators. Natural selection should favour the evolution of plant traits that deter ants from visiting flowers during anthesis, without waiving their defensive services. The Distraction Hypothesis posits that rewarding ants with extrafloral nectar could reduce their visitation of flowers, reducing ant‐pollinator conflict while retaining protection of other structures.
2. We characterised the proportion of flowers occupied by ants and the number of ants per flower in a Mexican ant‐plant, *Turnera velutina*. We clogged extrafloral nectaries on field plants and observed the effects on patrolling ants, pollinators and ants inside flowers, and quantified the effects on plant fitness. Based on the Distraction Hypothesis, we predicted that preventing extrafloral nectar secretion should result in fewer ants active at extrafloral nectaries, more ants inside flowers and a higher proportion of flowers occupied by ants, leading to ant‐pollinator conflict, with reduced pollinator visitation and reduced plant fitness.
3. Overall ant activity inside flowers was low. Preventing extrafloral nectar secretion through clogging reduced the number of ants patrolling extrafloral nectaries, significantly increased the proportion of flowers occupied by ants from 6.1% to 9.7%, and reduced plant reproductive output through a 12% increase in the probability of fruit abortion. No change in the numbers of ants or pollinators inside flowers was observed. This is the first support for the Distraction Hypothesis obtained under field conditions, showing ecological and plant fitness benefits of the distracting function of extrafloral nectar during anthesis.
4. *Synthesis.* Our study provides the first field experimental support for the Distraction Hypothesis, suggesting that extrafloral nectaries located close to flowers may bribe ants away from reproductive structures during the crucial pollination period, reducing the probability of ant occupation of flowers, reducing ant‐pollinator conflict and increasing plant reproductive success.

## 1 INTRODUCTION

Ant‐plants recruit ants by providing nesting sites and/food resources, and benefit from ant‐mediated reduction in damage by herbivores and pathogens (Bentley1977a; Chamberlain & Holland, 2009; Herrera & Pellmyr, 2009; Janzen, 1966; Rosumek et al., 2009; Trager et al., 2010). The most widespread reward produced by ant‐plants is extrafloral nectar (EFN), a key food resource for ants (Ochoa Sánchez, 2016; Rudgers & Gardener, 2004) that increases individual survivorship (Fisher, Sternberg, & Price, 1990), colony growth rate and reproductive output (Byk & Del‐Claro, 2011). Guarded plants in turn show increased somatic growth (biomass or leaf production) and reproductive output (Chamberlain & Holland, 2009; Escalante‐Pérez & Heil, 2012; Heil, Brigitte, Ulrich, & Linsenmair, 2001; Rosumek et al., 2009; Trager et al., 2010).

Most ant‐plants are angiosperms (Keeler, 2014), and many require the services of animal pollen vectors for seed set (Ballantyne & Willmer, 2012, Dutton et al., 2016, Villamil, Boege, & Stone, 2018, Bentley1977b, Torres‐Hernández, & Rico‐Gray, 2000, Díaz‐Castelazo, Rico‐Gray, Ortega, & Angeles, 2005, Rico‐Gray & Oliveira, 2007, Raine, Willmer, & Stone, 2002, among many other studies documenting animal‐pollinated ant‐plants), making ants and pollinators likely to co‐occur on a given host plant. This raises the possibility of several types of direct and/or indirect conflicts between ants and pollinators. First, an indirect conflict can arise if there is a trade‐off between plant allocation of resources to reproduction (which benefits pollinators) versus investment in growth and defence (which benefits ant guards) (Bazzaz, Chiariello, Coley, & Pitelka, 1987). Plants that do not reproduce grow faster and develop larger resource‐acquiring and producing organs (roots and leaves) (Frederickson, 2009), leading to indirect conflict between ants and pollinators over plant resources and rewards (Afkhami, Rudgers, & Stachowicz, 2014; Dutton et al., 2016). In extreme cases of ant‐pollinator conflict, ants actively increase plant investment towards growth and defence by castrating their host plant through consumption of floral meristems (Frederickson, 2009; Palmer et al., 2010) or mature inflorescences (Izzo & Vasconcelos, 2002). Second, ants may enter flowers and consume floral nectar without providing pollination services, providing no benefit to the plant and potentially reducing the attractiveness of flowers to effective pollinators (Rico‐Gray & Oliveira, 2007). Third, ant visits to flowers may reduce pollen viability by depositing antimicrobial substances that decrease pollen germination rates, and hence decrease male fitness for the plant (Dutton & Frederickson, 2012; Wagner, 2000). Finally, ants may attack or intimidate pollinators directly (Villamil et al., 2018; Wagner & Kay, 2002; Willmer et al., 2009), reducing flower visitation rates (Lach, 2008; Ness, 2006) or duration (Villamil et al., 2018). One hundred and forty years ago, Anton Joseph Kerner, an Austro‐Hungarian botanist, wrote:

Of all the wingless insects it is the widely dispersed ants that are most unwelcome guests to flowers. And yet are they the very ones which have the greatest longing for the nectar, as numberless observations sufficiently show. (Kerner, 1878, p. 21)

While ants may be unbidden floral visitors, they are also effective bodyguards (Bentley1977a,; Chamberlain & Holland, 2009; Rosumek et al., 2009; Trager et al., 2010), which may represent an ecological trade‐off for ant‐plants. Given that ant guards can have both costs and benefits for different aspects of plant fitness, we expect natural selection to act on ant‐plant traits to minimise the negative impacts of ants relative to the protection they provide, ameliorating the negative consequences of ant‐pollinator antagonism for plant fitness (Raine et al., 2002). A wide range of mechanisms have been interpreted as achieving this by reducing ant visitation to flowers during anthesis, including physical barriers (Carlson & Harms, 2007; Galen, 1999; Galen & Cuba, 2001; Harley, 1991; Raine et al., 2002; Willmer, 2011), chemical repellents (Agarwal & Rastogi, 2008; Ballantyne & Willmer, 2012; Junker & Blüthgen, 2008; Junker, Chung, & Blüthgen, 2007; Willmer et al., 2009; Willmer & Stone, 1997) or bribes (Kerner, 1878; Martínez‐Bauer, Martínez, Murphy, & Burd, 2015; Willmer, 2011). Physical barriers include spiny or hairy surfaces on the outside of the corolla or on floral pedicels that prevent tarsi from gripping and so hinder ant walking (Willmer, 2011), and waxy or sticky plant secretions that prevent ants from climbing (Harley, 1991). Bracts around the calyx can act as a water trap, creating a pool of water or mucilage that prevents ants and other small insects from crawling into the flowers (Carlson & Harms, 2007). The shape of the flower may itself stop ants from entering flowers: pendant, thin and constricted corollas are effective ant‐excluding morphologies (Galen, 1999; Galen & Cuba, 2001; Willmer et al., 2009). Several species produce ant‐repelling flowers (Agarwal & Rastogi, 2008; Junker & Blüthgen, 2008; Junker et al., 2007; Willmer et al., 2009) and furthermore, ant repellence may be concentrated in specific floral parts such as petals (Ballantyne & Willmer, 2012; Ness, 2006), or pollen and anthers (Ballantyne & Willmer, 2012; Ghazoul, 2001; Raine et al., 2002; Willmer et al., 2009; Willmer & Stone, 1997). Finally, some species may entice ants away from flowers by offering alternative sugary rewards outside the flowers, using EFN as a distraction or bribe (Chamberlain & Holland, 2008; Galen, 2005; Wagner & Kay, 2002; Willmer, 2011). During the nineteenth century, Kerner (1878) suggested that EFN in plants with floral nectar might serve to distract ants from visiting the flowers.

Any insects that creep along the stem must, if they would get at the flower, of necessity pass over this disk with its drop of nectar; thus what they would have found, in the flower, is already offered to them here in rich abundance. The creeping insects are not fastidious. They are content with that which is first offered, and so do not trouble themselves to climb farther up to the flowers. […] I do not therefore hesitate to interpret all nectar‐glands that are found on leaves as means of protection against the unwelcome, because unprofitable, visits of creeping insects. (Kerner, 1878, pp. 137–139)

The idea that EFN could distract non‐pollinating insects away from flowers and so reduce any disruption of pollination is known as the Distraction Hypothesis (Chamberlain & Holland, 2008; Holland, Scott, & Tom, 2011; Wagner & Kay, 2002). In many ant‐plants, flowers and extrafloral nectaries are in close proximity (Keeler, 2014; Weber & Keeler, 2013) and several species secrete extrafloral nectar only or predominantly during the flowering period (Bentley1977b,; Chamberlain & Holland, 2008; Dutton et al., 2016; Falcão, Dáttilo, & Izzo, 2014; Holland, Chamberlain, & Horn, 2010; Villamil, 2017; Villamil, Márquez‐Guzmán, & Boege, 2013; Villamil‐Buenrostro, 2012). For example, extrafloral nectaries on leaves associated with flowers of the Mexican ant‐plant *Turnera velutina* (Passifloraceae) secrete more nectar with higher sugar content than extrafloral nectaries on leaves bearing buds and fruits (Villamil, 2017). This increase in EFN secretion during anthesis is compatible with the Distraction Hypothesis, in that EFN secretion near flowers could lure and bribe ants that might otherwise enter flowers seeking floral nectar. However, the same floral behaviour and the frequent proximity of extrafloral nectaries to reproductive structures can also be explained by the Optimal Defence Theory (ODT), which predicts that plants should focus defensive investment on highly vulnerable and valuable tissues for plant fitness, such as flowers, fruits and seeds (Stamp, 2003). Finally, it is possible that high EFN secretion on flowering shoots in myrmecophiles could fulfil both distracting and protective roles, simultaneously keeping ants out of flowers but promoting their patrolling around reproductive tissues to deter herbivores.

While the defensive role of ant recruitment through EFN secretion has been widely demonstrated (Chamberlain & Holland, 2009; Rosumek et al., 2009; Trager et al., 2010), the Distraction Hypothesis has not been adequately tested. To our knowledge, since Kerner proposed it in 1878, only three experimental studies have been performed and all have rejected it (Chamberlain & Holland, 2008; Galen, 2005; Wagner & Kay, 2002). However, none of these studies were carried out in an ecologically realistic setting (a point that we address further in the Discussion).

Here we use experimental manipulation of EFN secretion during anthesis in a Mexican endemic plant, *T. velutina*, to test the Distraction Hypothesis under natural conditions, improving on previously reported experimental designs. We evaluated the potential ecological and fitness consequences of the Distraction Hypothesis, addressing the following questions: (a) How often are flowers occupied by ants and how many ants are found in them? (b) Does preventing EFN secretion affect the number of ants patrolling extrafloral nectaries, the number of ants inside the flowers or the number of pollinators visiting flowers? (c) Does preventing EFN secretion increase the probability of a flower being occupied by ants? (d) Does the number of ants at extrafloral nectaries or inside flowers affect pollinator visitation? (e) Does preventing EFN secretion affect plant fitness? If the Distraction Hypothesis is true, we predict that experimental elimination of extrafloral nectar secretion should: (a) reduce ant visitation to extrafloral nectaries, (b) increase the numbers of ants inside flowers, (c) increase the proportion of flowers occupied by ants, (d) leading to decreased levels of floral visitation by pollinators and (e) a reduction in plant fitness.

## 2 MATERIALS AND METHODS

### 2.1 Study site and system

**Figure 1:**
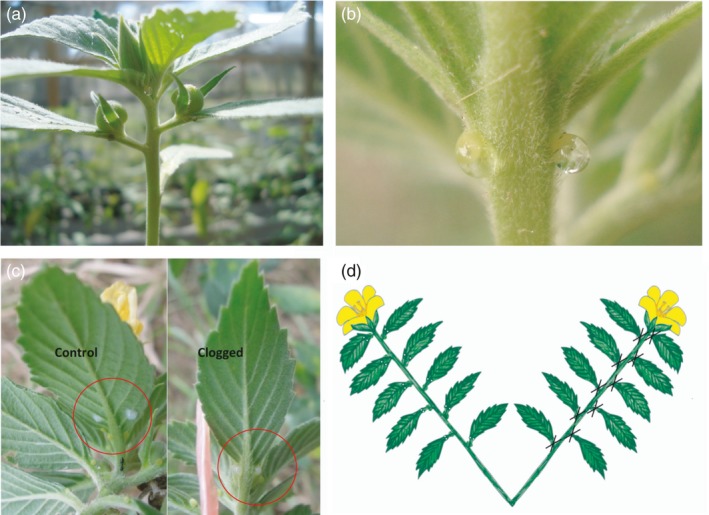
Images showing (a) an apex of *Turnera velutina* bearing an apical flower bud and two lateral fruits, (b) the location of extrafloral nectaries on the underside of a leaf, (c) a comparison of clogged and control leaves and (d) the spatial arrangement of the long‐term experiment with black crosses indicating clogged extrafloral nectaries

All experiments and observations were conducted in the stabilised coastal sand dunes at the CICOLMA Field Station in La Mancha, Veracruz, in the Gulf of Mexico. Within this population, we selected four sites with high densities of *T. velutina*(Passifloraceae), a myrmecophile (Cuautle & Rico‐Gray, 2003) Mexican endemic perennial shrub (Arbo, 2005). At La Mancha, *T. velutina*establishes a facultative mutualism with at least 13 ant species (Cuautle, Rico‐Gray, & Díaz‐Castelazo, 2005; Zedillo‐Avelleyra, 2017) and its main herbivores are caterpillars of a butterfly, *Euptoieta hegesia*(Nymphalidae). Extrafloral nectar is provided in paired cup‐shaped glands located on the underside of the leaf blade or petiole (Figure 1). Although it flowers year‐round, flowering peaks during summer (Cuautle et al., 2005). Flowers last 1 day, are insect‐pollinated (Sosenski, Ramos, Domínguez, Boege, & Fornoni, 2016) and have a yellow, pentamerous, campanulate corolla with nectar easily accessible at the base. Honeybees (*Apis mellifera*) are the dominant pollinators at La Mancha, accounting for 94% of visits (Sosenski et al., 2016; Villamil et al., 2018).

### 2.2 Fieldwork methods

#### 2.2.1 Surveys of ants inside flowers

We quantified ant occupancy in flowers of *T. velutina* by surveying 1,604 flowers across four sites within CICOLMA in November 2014. Flowers at each site were observed and instant counts were recorded every hour throughout the whole anthesis period (08:30–12:30 hr), with one observer at each site. We estimated the proportion of flowers occupied by ants, and the total number of ants across occupied flowers within a site. Flowers were sampled at the same site over multiple days, for 10 site‐and‐day combinations. Since these are 1‐day flowers, we considered each site‐day as a replicate (*n = *10 site‐days), with site‐and‐day effects incorporated into our statistical modelling (see below).

#### 2.2.2 Experimental manipulation of EFN secretion

To test the Distraction Hypothesis, we experimentally clogged extrafloral nectaries to prevent nectar secretion and compared ant and pollinator behaviours on paired shoots with and without EFN secretion. This experiment was conducted over 5 days during November 2014. Early on each day of the experiment, a pair of neighbouring, unopened floral buds within a plant were marked as either control or clogged treatments (*n* = 216 flowers; *n* = 108 pairs, *n* = 108 plants). EFN secretion on clogged treatment leaves was eliminated by sealing the nectary cup with a droplet of transparent acrylic textile paint (Mylin dimensional, Mexico). On control treatment leaves, we applied similarly sized droplets of the same textile paint a couple of millimetres above the gland (Figure 1), controlling for any effects of the acrylic paint itself. Pilot tests confirmed that the paint totally prevented EFN secretion and also that the paint did not deter ants or pollinators. We recorded the frequency and identity of ants (to genera or species level following Zedillo‐Avelleyra, 2017) and other insects visiting each flower pair and the associated extrafloral nectaries for 2 min every hour during anthesis (08:30–12:30 hr). Simultaneous observations were performed at each of three sites by different observers. For brevity, we refer to non‐ant flower visitors as pollinators, while recognising that the efficacy of visits by all species mentioned in contributing to seed set in *T. velutina*remains to be demonstrated.

Based on the results from the clogging experiment described above (from now on referred to as the short‐term experiment), we conducted a follow‐up experiment in which treatment duration and spatial scale were both increased by a factor of 10, using paired branches and focusing on one flower on each control or clogged branch, rather than paired flowers on the same branch. We refer to this experiment from now on as the long‐term experiment (see Supporting Information 1 for further details). The extrafloral nectaries of all 10 leaves on the clogged treatment branches were sealed as described above, and the treatment was maintained for 10 days (Figure 1). Our hypothesis was that increasing both the temporal and spatial scales of our treatment would result in a larger experimental effect size. However, a comparison of the results from the short‐term and long‐term experiments showed that ants respond at a smaller scale (Supporting Information 1: Table S2), and we therefore focus on the results of the short‐term experiment and highlight differences in results for the longer term, larger scale experiment where these are relevant to the Distraction Hypothesis. Full results and details regarding the long‐term experiment are provided in Supporting Information 1.

#### 2.2.3 Impacts of EFN secretion on fitness

To quantify the impact of clogging EFN secretion and the Distraction Hypothesis on plant fitness, we collected the fruits resulting from experimental flowers (control and clogged) at which pollinator visitation was observed. We recorded whether those flowers developed into fruits with seeds or whether they were aborted, and counted the number of seeds per fruit. All fruits were collected at least 1 week post‐anthesis, at which stage retained fruits can be distinguished from aborted fruits, and developing seeds can be counted distinguishing viable from unviable seeds, even if still immature.

### 2.3 Statistical analyses

All statistical analyses were conducted in r version 3.23 (R Core Team, 2014). Mixed effects models were fitted using “lme4” (Bates, Maechler, Bolker, & Walker, 2015) or “MCMCglmm” (Hadfield, 2010) r packages.

#### 2.3.1 Surveys of ants in flowers

To test if the proportion of flowers with ants inside them changed over the anthesis period, we fitted a binomial mixed model with time of day as a fixed effect. Flowers of *T. velutina*last for a single day, and because multiple flowers were sampled on a given site on a given day, we fitted site identity as a random effect to account for differences between site‐and‐day variation in variables that could influence ant abundance, such as resource availability, ant diversity, or the abundance of ant nests. Tukey post hoc comparisons were used to test differences between hours using the “multcomp” r package (Hothorn, Bretz, & Westfall, 2008).

To test if the number of ants inside occupied flowers changed over the anthesis period, we fitted a Poisson mixed model, using the number of ants inside flowers per site as the response variable and fitted as fixed effects time of day as a linear and as a quadratic term. The number of flowers occupied by ants was fitted as a log‐transformed offset to control for ant density in flowers, which is likely to decrease in sites with more flowers occupied by ants, since we recorded counts per site rather than counts per individual flower (see fieldwork methods). Time of day was fitted as a linear and as a quadratic term to investigate the shape of the activity pattern of ants in flowers relationship between the number of ants inside flowers through the day. We fitted site identity as a random effect to account for variation that could influence ant abundance (as detailed above). We also included an observation‐level random effect where each data point receives a unique level of a random effect to control for overdispersion (Hinde, 1982). Tukey post hoc comparisons were used to test differences between hours using the “multcomp” r package (Hothorn et al., 2008).

#### 2.3.2 Ecological consequences of EFN secretion

Five mixed effects models (i‐v) were fitted to test the ecological consequences of the Distraction Hypothesis. Because all of these models had the same random effects structure unless otherwise specified, we detail the random effects first and then describe the fixed effects for each model. Flower identity was fitted as a random effect to account for repeated hourly observations. Because this experiment had a paired experimental design, we fitted flower pair identity as a random effect to control for between‐pair variation in floral and extrafloral investment. We also included an observation‐level random effect where each data point received a unique level of a random effect to control for overdispersion. We fitted the following models, and have structured our results following the same order:

- To test the effect of nectary clogging on the number of ants we fitted a Poisson mixed effects model using number of ants as the response variable. Ant location (at extrafloral nectaries or in flowers), treatment and the interaction between these two factors were fitted as fixed effects. Tukey tests were conducted to test differences between the number of ants at extrafloral nectaries or flowers under control or clogged gland conditions.
- To test whether preventing EFN secretion by clogging the glands increased the probability of flower occupancy by ants, we fitted a binomial mixed effect model with the presence or absence of ants in a flower as a response variable. Clogging treatment was fitted as a fixed effect. The observation‐level random effect was omitted.
- To test the effect of clogging EFN secretion on pollinator visitation, we fitted a Poisson mixed model using the number of pollinators as the response variable and treatment as the only fixed effect.
- To test the effect that the total number of ants had on pollinator visitation (regardless of their location in flowers or at extrafloral nectaries), we fitted a Poisson mixed model using number of pollinators as the response variable. As fixed effects we fitted the total number of ants, and treatment to test whether treatment affected pollinator visitation in a way that was unlinked to the number of ants.
- To test if the location (inside flowers or at extrafloral nectaries) and number of ants had an effect on pollinator visitation, we fitted a Poisson mixed model. The number of pollinators was fitted as the response variable, while treatment, number of ants in flowers, and number of ants at extrafloral nectaries were fitted as fixed effects.

Data from the long‐term experiment were analysed following a similar model structure reported for the ecological consequences models (S.i‐v, see Supporting Information).

#### 2.3.3 Impacts of EFN secretion on plant fitness

- To test the effect of clogging on fruit abortion rates, we fitted a binomial mixed model, with clogging treatment as the fixed effect and pair identity as a random effect.

- For those fruits that developed seeds, we tested the effect of clogging on the number of seeds by fitting a Poisson mixed model. Clogging was fitted as a fixed effect and as random effects we fitted pair identity and an observation‐level random effect to account for overdispersion.

#### 2.3.4 Exploring the responses and effects of different ant species

We fitted additional models aiming to explore differences between ant species in their response to clogging and in their effects on pollinator visitation. We investigated whether ant species differed in their response to clogging (see model S.vi in Supporting Information), and whether different ant species patrolling the plants and/or inside the flowers differed in their effect on pollinator visitation (see models S.vii, S.viii in Supporting Information). These models allowed us to: (a) estimate the effects of individual ant species on pollinator visitation, (b) account for plants occupied by multiple ant species and (c) capture the variation in ant abundance within a given ant species.

#### 2.3.5 Effect sizes

Cohen *d*effect sizes for all models were calculated using the likelihood ratio tests (LRT) statistics from each model. To test whether increasing the duration and scale of the clogging treatment by a factor of 10 had a larger effect on the number of ants and pollinators, we estimated the ratio of change in the effect size between the short‐ and long‐term experiment for each type of visitor (See Supporting Information).

## 3 RESULTS

### 3.1 Surveys of ants in flowers

We observed 10 ant species from four subfamilies interacting with *T. velutina*: *Dorymyrmex bicolor*(Dolichoderinae), *Camponotus planatus*, *Camponotus mucronatus*, *Camponotus novogranadensis*, *Brachymyrmex* sp. and *Paractrechina longicornis*(Formicinae); *Cephalotes*sp., *Crematogaster*sp. and *Monomorium ebenimum*(Myrmicinae); and *Pseudomyrmex gracilis*(Pseudomyrmicinae). We observed that ants associated with plants vary spatially, and we assume this is due to variation in the proximity of nests of different ant species. Though not formally quantified, we observed apparent differences in ant behaviour among species. Some species patrolled individually, such as *Camponotus planatus*, *C. mucronatus* and *C. novogranadensis*, while others were gregarious, such as *D. bicolor*, *Brachymyrmex* sp., and *Paratrechina longicornis*. *Monomorium ebenimum* probably provides no guarding services to *T. velutina* since they have only been observed consuming floral nectar and not patrolling elsewhere. In addition, this species belongs to a world‐wide genus of floral nectar thieves (Bolton, 1987; Ettershank, 1966). Feeding preferences also vary among ant species, from opportunistic carnivores such as *Pseudomyrmex gracilis* found inside flowers hunting for thrips and beetles, to omnivores such as *Crematogaster*sp. that harvest elaiosomes attached to *T. velutina*′s seeds (S. Ochoa‐López, pers. comm., Dec. 2014).

**Figure 2:**
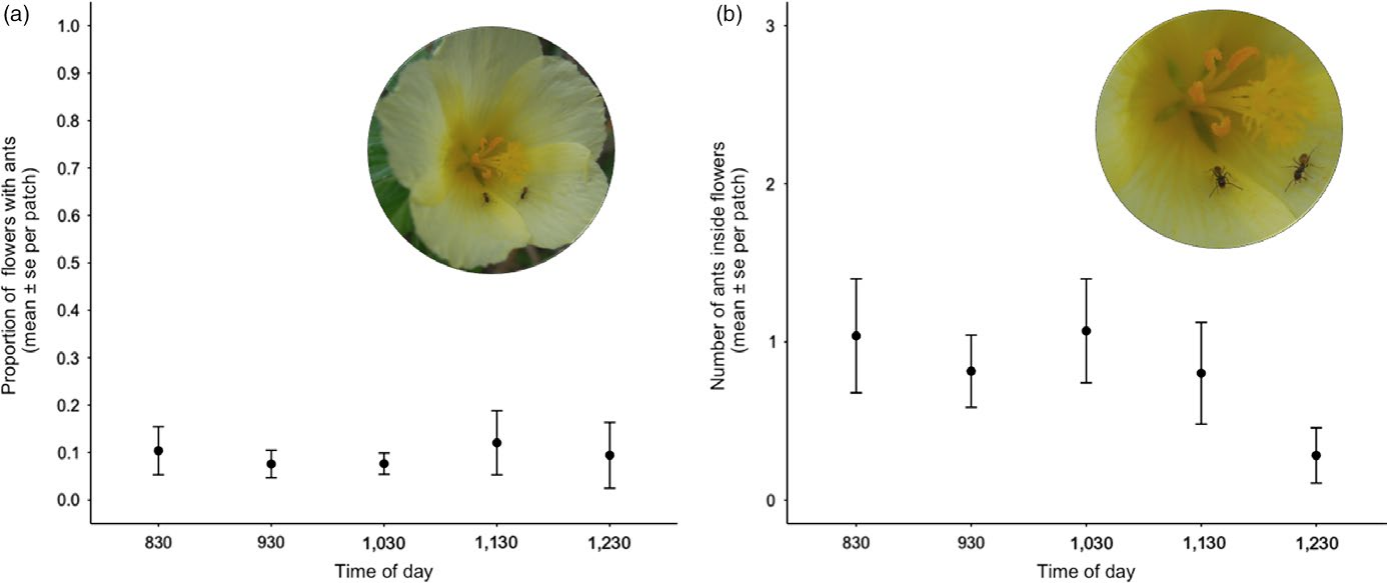
(a) Proportion of flowers with ants inside them and (b) number of ants per flower throughout the anthesis period (mean ± *SE* per site) in hourly observations (*n = *42 observations, from 10 sites)

Across all four sites and over all time intervals, surveys of the frequency and abundance of ants inside flowers revealed that 9.30 ± 0.19% of the flowers within a site were occupied by ants, with an average density of 2 ± 0.28 ants/occupied flower. The low proportion of flowers (Figure 2a) with low numbers of ants (Figure 2b) was constant throughout the anthesis period (Table 1). The number of ants inside flowers did not vary significantly through daily time and we found no statistical support for any quadratic effect.

**Table 1:** Estimates and likelihood ratio test results for statistical models used to test ant occupation of flowers, and the ecological and plant fitness consequences of clogging extrafloral nectar (EFN) secretion on *Turnera velutina*. Ant location stands for the number of ants patrolling EFN or inside flowers. The values highlighted in bold are statistically significant (*p < 0.05; ***p < 0.001; NS = non‐significant, *p* > 0.05), OLRE stands for observation‐level random effect

Experiment	Model	Response	Fixed effects	Estimate	LRT	*P*‐value	Random effects	Variance	*SD*
Surveys of ants in flowers		Proportion of flowers with ants inside	Time of day	−0.0127	−0.017	0.98	Site	0.29	0.53
		Number of ants per flower	log (Flowers with ants)	0.6362	18.55	1.64^−05^***	Site OLRE	5.09^−09^ 0.20	0 0.45
			Time of day	0.3288	1.54	0.21			
			Time of day^2^	−0.0771	1.12	0.28			
Clogging: Ecological consequences	i)	Number of ants	Clogging	−0.5314	12.42	0.0004***	Flower Pair OLRE	0.39 1.04 0.28	0.62 1.01 0.53
			Ant location	−0.2772	647.09	2.2^−16^***			
			Clogging × ant location	0.7656	13.28	0.0002***			
	ii)	Proportion of flower occupied by ants	Clogging	0.7669	4.61	0.031*	Flower Pair	0.38 5.81	0.62 2.41
	iii)	Number of pollinators	Clogging	−0.0749	0.33	0.56	Flower Pair OLRE	0 0.89 0.01	0 0.94 0.12
	iv)	Number of ants inside flowers	Clogging	0.4413	3.41	**0.06**	Flower Pair OLRE	2.02^−08^ 4.36 0.72	1.42^−05^ 2.08 0.85
	iv)	Number of pollinators	Clogging	−0.0947	0.52	0.46	Flower Pair OLRE	0 0.86 0.01	0 0.92 0.13
			Total ants	−0.0569	1.97	0.16			
	v)	Number of pollinators	Clogging	−0.1336	1.01	0.31	Flower Pair OLRE	1.53^−8^ 0.83 0.019	1.23^−05^ 0.91 0.13
			Ants at EFN	−0.0967	4.20	0.04*			
			Ants in flowers	0.1897	1.63	0.20			
	vi)	Number of pollinators	Clogging	−0.1848		0.220	Flower Pair Ant species	0.023 1.228 0.020	
			Ants at EFN	−0.1219		0.099			
			Ants in flowers	−0.1309		0.512			
Clogging: Impacts on plant fitness	vi)	Fruit abortion	Clogging	0.6059	3.54	0.05*	Pair	4^−14^	2^−7^
	vii)	Number of seeds	Clogging	−0.1512	1.241	0.265	Pair OLRE	0.12 0.84	0.35 0.92

### 3.2 Floral visitors

All but one of 202 visits to *T. velutina*flowers by non‐ant visitors were made by other insects (Table 3). The only exception was a single visit by a hummingbird (Trochilidae). Of the insect visits, 90.5% were by honeybees, *A. mellifera*, with most of the remainder visits being by native bees and butterflies.

### 3.3 Ecological consequences of EFN removal

**Figure 3:**
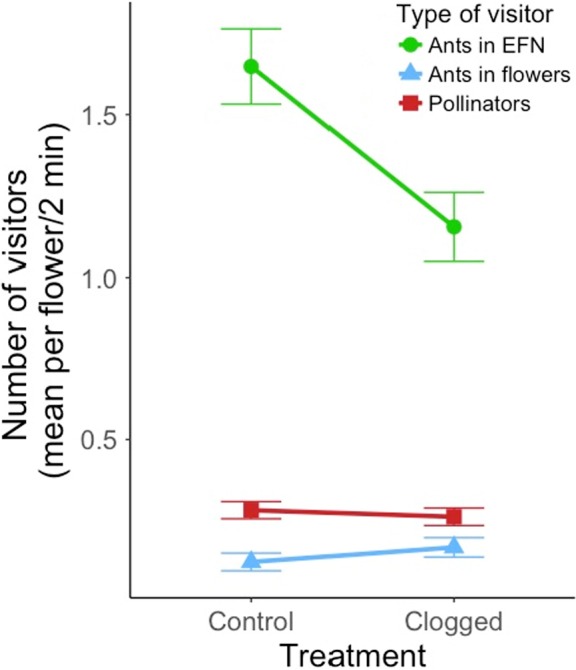
Mean numbers of visitors to flowers of *Turnera velutina* (mean ± 1 *SE*) recorded in hourly surveys during 2 min of observation per flower for the short‐term experiment. Clogged treatment flowers had secretion of extrafloral nectar (EFN) prevented by clogging the associated extrafloral nectaries. The short‐term experiment involved prevention of EFN secretion associated with one flower for 1 day (see Figure 1). Red circles represent ants at extrafloral nectaries; blue triangles represent ant in flowers, and green squares represent pollinators

Numbers of patrolling ants were significantly affected by EFN treatment (clogged vs. control), ant location and the interaction between these factors (Figure 3a; Table 1). Ten times more ants were found patrolling extrafloral nectaries (1.49 ± 0.079 ants) than were found inside flowers (0.14 ± 0.02 ants) (Figure 3a; Table 1), regardless of treatment (control: *Z* = −17.23, *p* < 0.001; clogged: *Z* = −14.03, *p* < 0.001). The effect of eliminating EFN secretion on the number of ants differed between extrafloral nectaries and flowers, resulting in a significant decline in numbers of ants patrolling extrafloral nectaries (*Z* = −4.22, *p* < 0.001), but no significant change in the numbers of ants observed inside flowers (*Z* = 1.05, *p* = 0.705; Figure 3a; Table 1). The percentage of flowers occupied by ants increased significantly from 6.1% under the control treatment to 9.7% when extrafloral nectaries were clogged (Table 1).

Numbers of flower visitors were not significantly affected by the elimination of EFN secretion (Figure 3a; Table 1), nor was there any significant interaction between visitor numbers and the total number of ants (Table 1). When ant abundance was partitioned by location on the plant (at extrafloral nectaries or in flowers), neither the number of ants patrolling extrafloral nectaries nor the number of ants inside a flower had a significant effect on the number of flower visitors (Table 1).

In all five models used to analyse the short‐term experiment (one leaf, 1 day), differences between individual plants (captured by the pair random effect) explained the largest proportion of variation in the numbers of ants and pollinators (Table 1). Differences between individual flowers (captured by the flower random effect) or random variation between observations (captured by the OLRE random effect) explained smaller proportions of variation in the numbers of ants or pollinators (Table 1).

### 3.4 Impacts of EFN secretion on plant fitness

**Figure 4:**
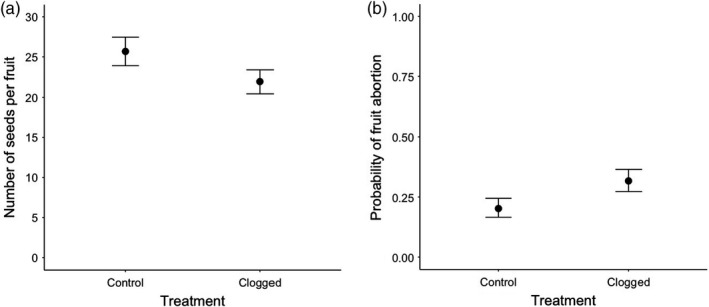
Effects of clogging the extrafloral nectaries on (a) the number of seeds (mean ± *SE*) produced by *Turnera velutina* and (b) the probability of fruit abortion (mean ± *SE*)

Clogging had a marginally significant effect (*p* = 0.059) on fruit abortion, increasing by 12% the probability of abortion in flowers associated with leaves in which EFN had been clogged (Figure 4a, Table 1). Despite the *p‐*value being marginally significant, clogging had a considerable Cohen *d*effect size (Cohen, 1988) on fruit abortion (Table 2). However, clogging had no effect on the number of seeds per fruit (Figure 4b, Table 1) with a small Cohen *d*effect size between treatments (Cohen, 1988) (Table 2).

**Table 2:** Cohen *d* effect sizes in the short‐term clogging experiment for the number of visits per visitor type and plant fitness consequences. Magnitudes of effect sizes are defined according to Cohen (1988)

Model	Short‐term experiment: Clogging 1 day 1 leaf		
	Response	*d*	Effect size
Ecological consequences			
i)	Ants at EFN	−0.2865	Small
i)	Ants in flowers	+0.070	ns
ii)	Flowers occupied by ants	+0.146	Small
iii)	Pollinators	+0.0503	ns
iv)	Clogging	−0.0488	ns
iv)	Total ants	−0.0915	ns
v)	Clogging	−0.0676	ns
v)	Ants at EFN	−0.1314	ns
v)	Ants in flowers	−0.0893	ns
Fitness consequences			
vi)	Fruit abortion	+0.5185	Medium
vii)	Seeds	−0.1565	ns

### 3.5 Comparison of patterns across spatio‐temporal scales

In contrast to our prediction, increasing the duration and spatial scale of the clogging treatment by a factor of 10 did not result in larger effect sizes on ant behaviours (Table S2). In fact, the long‐term clogging experiment had less impact on ant patrolling than the short‐term clogging experiment, resulting in smaller effect sizes on numbers of ants at extrafloral nectaries, ants inside flowers and on the proportion of flower occupancy by ants (Table S2). The impact of preventing EFN secretion on the number of ants inside flowers changed from positive at a short‐term, local scale to negative in the long‐term, branch‐scale experiment (10 leaves, 10 days) (Table S2).

### 3.6 Ant species‐specific responses to clogging and effects on pollinators

Although ant species explained only 0.21% of the variation in the number of ants inside flowers (Model S.vi in Table S3), there was variation between ant species in responses to clogging (Figure S3a). *Brachymyrmex*sp. ants were the most abundant ants found inside flowers, as shown by the non‐zero‐overlapping effect (Figure S3a, Table 3). While effect estimates vary for other ant species, confidence intervals for all taxa other than *Brachymyrmex*sp. overlap with zero (see Table 3 for rank order of abundance inside flowers and Figure S3a for likelihood of response to clogging; see Supporting Information for further details). Activity by individual ant taxa at extrafloral nectaries had very small effects on pollinator visitation, as shown by the small estimates (model S.vii, Table S4), although their effects were precisely estimated by our models, as indicated by narrow variation around these estimates (Figure S3b). In contrast, the effects of activity by individual ant taxa inside flowers on pollinator visitation could not be precisely estimated from our data, as indicated by the large variation associated with these estimates (model S.viii, Figure S3c).

**Table 3:** Taxonomic identities of floral visitors recorded in the short‐term clogging experiment. Taxa with the epithet “sp.” were identified only to genus, but all the individuals belong to the same morphospecies

Taxon	Number of visitors		Subfamily
	At EFN	In flowers	
Ants at EFN			
*Dorymyrmex bicolor*	373	10	Dolichoderinae
*Brachymyrmex*sp.	342	74	Formicinae
*Paratrechina longicornis*	166	3	Formicinae
*Camponotus planatus*	128	1	Formicinae
*Camponotus mucronatus*	41	4	Formicinae
*Camponotus*sp.	49	5	Formicinae
*Camponotus novogranadensis*	3	1	Formicinae
*Crematogaster*sp.	26	1	Myrmicinae
*Cephalotes*sp.	16	0	Myrmicinae
*Monomorium ebenium*	58	11	Myrmicinae
*Pseudomyrmex gracilis*	18	0	Pseudomyrmicinae
Unidentified ants	13	0	?
Floral visitors			
*Apis mellifera*		183	
Native bees (Apoidea)		12	
Diptera		1	
Lepidoptera		4	
Wasps		1	
Hummingbird		1	

## 4 DISCUSSION

### 4.1 The distraction hypothesis

Plants face a potential trade‐off between the benefits they receive from ants patrolling their leaves and flowers and the costs associated with this activity (Altshuler, 1999; Assunção, Torezan‐Silingardi, & Del‐Claro, 2014; Dutton et al., 2016). In *T. velutina*, the presence of the most aggressive ants inside flowers increases the likelihood of pollinators displaying alert behaviours and reduces the time honeybees spend inside the flowers (Villamil et al., 2018). To reduce the costs without waiving the protective benefits, several authors have hypothesised that plants should evolve mechanisms that minimise ant access to floral structures and pollinators, while recruiting them to the vicinity in order to reduce herbivore damage (Martínez‐Bauer et al., 2015; Willmer & Stone, 1997). Two current theories—the Distraction Hypothesis and ODT—are compatible with the commonly observed location of extrafloral nectaries close to valuable and vulnerable reproductive structures. The Distraction Hypothesis specifically predicts that EFN secretion draws ant guards away from flowers in such a way that ant‐pollinator conflict is reduced (Kerner, 1878). The Distraction Hypothesis has been widely overlooked, with only three studies addressing it since its proposal in 1878. We briefly outline these studies below, highlighting aspects of their experimental design that contrast with our approach, and we summarise the extent to which our results match predictions of the Distraction Hypothesis and ODT.

Wagner and Kay (2002) tested the Distraction Hypothesis using sticks as artificial plants, and identical plastic caps as artificial floral (primary) or extrafloral (additional) nectaries. Sticks with additional nectar sources did not attract more ants, but reduced the number of ants at primary sources. They concluded that additional (extrafloral) nectar sources did not increase ant recruitment, but distracted ants from the primary, floral nectar sources (Wagner & Kay, 2002). These results differ from studies conducted on natural plants (Bentley, 1976; Shenoy, Radhika, Satish, & Borges, 2012; Villamil et al., 2013) and from our findings (Figure 3), which show that increased EFN results in increased ant visitation. Furthermore, the plastic caps used by Wagner and Kay (2002) to simulate floral (primary) and extrafloral (additional) nectaries were morphologically identical and equally accessible, but neither assumption is met in natural EFN‐bearing species (Escalante‐Pérez & Heil, 2012; Keeler, 2014). Therefore, no robust conclusions about the Distraction Hypothesis can be drawn from this experimental design.

In 2005, Galen tested the Distraction Hypothesis on *Polemonium viscosum*, a plant species without extrafloral nectaries. Extrafloral nectaries were simulated by trimming the petals, anthers and pistils from some flowers, leaving only the calyx and toral disc that bears the floral nectaries to simulate extrafloral nectaries (Galen, 2005). Control inflorescences contained only intact flowers, while inflorescences with simulated extrafloral nectaries contained intact flowers plus trimmed flowers simulating extrafloral nectaries (Galen, 2005). Intact flowers in the EFN‐simulation inflorescences had higher ant visitation than flowers from control inflorescences, and Galen saw this result as rejecting the Distraction Hypothesis. However, rather than testing the Distraction Hypothesis, we suggest that this experiment tested the effect of total floral nectar availability on ant recruitment, and the effect of removing floral parts on ant visitation to flowers. By trimming the corolla and sexual organs, Galen facilitated ant access to the flower. Previous studies on *P. viscosum* demonstrated that corolla morphology effectively excludes ants from flowers (Galen, 1999; Galen & Cuba, 2001). Furthermore, artificial damage (trimming) is a confounding factor because it triggers plant‐induced defences (Ballaré, 2011; Heil, 2008; Heil, Koch et al., 2001; Ness, 2003) that strongly affect floral and extrafloral nectar secretion (Heil, 2011, 2015; Ness, 2003; Radhika, Kost, Bartram, Heil, & Boland, 2008). Consequently, higher ant visitation to intact flowers in the EFN‐simulation inflorescences may have been a response to the trimming of neighbouring flowers.

Finally, Chamberlain and Holland (2008) tested The Distraction Hypothesis on *Pachycereus schottii*, a senita cactus bearing extrafloral nectaries. They found higher rates of ant visitation on flowers from plants where EFN had been experimentally removed, as the Distraction Hypothesis would predict. However, in contrast to our clogging treatment in *T. velutina*, Chamberlain and Holland's EFN‐elimination treatment consisted of removing EFN secreting structures (buds, flowers and fruits). Hence, as in Galen's (2005) study, the increase in flower–ant interactions observed on EFN‐removal plants could be an ant response to the artificial damage inflicted by removing reproductive structures.

We tested the Distraction Hypothesis in a field population of *T. velutina*, a species bearing extrafloral nectaries, by experimentally manipulating EFN availability without inducing artificial damage to plant structures. If the Distraction Hypothesis is true and EFN distracts ants from entering the flowers, we predicted that elimination of EFN by clogging extrafloral nectaries should result in: (1) decreased numbers of ants patrolling extrafloral nectaries, (2) increased numbers of ants inside flowers, (3) an increase in the proportion of flowers occupied by ants, leading to (4) a reduction in the numbers of pollinators visiting the flowers, and (5) a reduction in plant fitness. If EFN secretion has evolved to reduce herbivore damage to flowers by increasing ant activity in their proximity, as predicted by ODT, then we expect elimination of EFN to result in patterns compatible with predictions 1 and 5 above, with the difference that reduced plant fitness should be caused by increased floral herbivory rather than ant‐associated reduction of visitation. However, ODT does not make predictions 2, 3 and 4.

Our results support the Distraction Hypothesis with predictions 1, 3 and 5 being met. We found that clogging EFN secretion reduced the number of ants patrolling extrafloral nectaries by 30% (prediction 1), increased the likelihood of flower occupation by ants by 3.6% (prediction 3), and increased the likelihood of fruit abortion by 12% (prediction 5). However, we found no significant increase in the number of ants inside flowers (prediction 2), or reduction in pollinator visitation (prediction 4) when extrafloral nectaries were clogged (Tables 1 and 2). Support for prediction 3 (increased flower occupation by ants), and reduction in plant fitness through increased rates of fruit abortion (rather than damage to flowers; Figure 4) are both specific to the Distraction Hypothesis. We therefore conclude that our results represent the first experimental support for this hypothesis obtained under field conditions.

### 4.2 Fitness consequences

The clogging treatment caused a 12% increase in the probability of fruit abortion, which is not linked to the visitation frequency as the number of pollinators was unchanged. We hypothesise this reduction in fitness when EFN was removed may be linked to changes in other aspects of pollinator visitation, such as a reduction in the duration of visits or changes in pollinator behaviours inside flowers which may have cascading effects on plant mating systems and pollen deposition patterns. However, further studies are required to assess the effect of ant patrolling on the duration of pollinator visits and behaviour. Shorter visits may result in reduced pollen deposition which may result in fruit abortion if ovules are not fertilised. Ant patrolling may also affect the plant mating system, affecting the selfing/outcrossing rates, which may lead to fruit abortion due to selective abortion linked to pollen origin or inbreeding depression. Plants can abort fruits with a higher proportion of selfed seeds, to increase resource allocation to fruits with a higher proportion of outcrossed seeds. Selective fruit abortion linked to pollen origin (selfing vs. outcrossing) has been observed in a wide array of plant species (Huth & Pellmyr, 2000; Marshall & Ellstrand, 1988; Niesenbaum, 1999; Stephenson, 1981).

### 4.3 Exploring ant species‐specific effects

We found 10 ant species interacting with *T. velutina*, representing a diverse mosaic of partners that may differ in their response to clogging and on their effects on pollinators. Evolution of plant mechanisms that reduce plant–pollinator conflict could be driven by interactions with one or more of these species. We expect plants to evolve phenotypes that favour ant taxa that are both effective guards and that have minimal net negative impacts, including interference with pollinators. Despite the potential for variation in effects across ant taxa, we found that ant species and the interaction between clogging and ant species had a negligible effect on the number of ants found inside flowers (Table S4) and on pollinator visitation (Table S4).

The estimates of the effects that individual ant species inside flowers have on pollinator visitation in model (S.vii) are imprecise (Figure S3c) for two main reasons: first, ants rarely occupy flowers (Figure 2, Table 1), and second, the scarce variation in ant species composition between flowers, caused by *Brachymyrmex*sp. being the dominant ant taxa inside flowers, resulting in few observations for other taxa (Table 3). For these two reasons, our data show little variation and model estimates are thus driven largely by uncertainty and further experiments are required to test the effect different ant species inside flowers have on pollinator visitation (Villamil et al., 2018). Finally, although models S.vi–S.viii do not elucidate the effects of specific ant species, they demonstrate quantitatively that lumping ant species together and testing the Distraction Hypothesis on the ant community associated with *T. velutina*is an adequate approach given the constraints of our dataset imposed by the biology of this system (see Supporting Information for further details).

Little is known about the extent to which positive and negative impacts of ant taxa are correlated and whether ant species that are threatening for herbivores (hence, highly defensive species) are also threatening for pollinators (hence, ecologically costly via pollinator deterrence) (but see: Ness, 2006, Miller, 2007, Ohm & Miller, 2014, LeVan, Hung, McCann, Ludka, & Holway, 2014, Villamil et al., 2018, Villamil et al., 2018). Large‐bodied and eusocial pollinators such as *A. mellifera* have been assumed to be less susceptible than smaller solitary bees or other non‐eusocial pollinators to ant attacks and more prone to visit flowers patrolled by aggressive ants (Brechbühl, Casas, & Bacher, 2010; Brechbühl, Kropf, & Bacher, 2010; Gadagkar, 1990; Queller, 1989; Romero, Antiqueira, & Koricheva, 2011).

Our findings suggest that ant species vary in their deterrent effect on *A. mellifera* bees (Figure S3). Qualitative patterns show that the presence of the most aggressive ant species, *D. bicolor*, inside flowers and at extrafloral nectaries have, on average, a negative effect on pollinators. These results are consistent with experimental findings demonstrating that placing dead *D. bicolor*ants inside flowers of *T. velutina*induced alert behaviours in *A. mellifera*, reduced visit duration and increased handling time per flower leading to a decrease in pollinator foraging efficiency (Villamil et al., 2018). However, *A. mellifera* honeybees are introduced pollinators, and further work is required to assess the effect of the ant community on pollinator assemblages dominated by native, smaller bodied, solitary pollinators.

### 4.4 The spatio‐temporal scale of the distraction hypothesis

Based on the relatively small effect sizes of the short‐term leaf‐scale experiment (Tables 1 and 2), we hypothesised that clogging the glands of only one leaf for 1 day was perhaps too local and short‐term a treatment to detect a measurable effect. In the long‐term experiment, we therefore increased both the spatial scale and duration of the EFN‐removal treatment by a factor of 10, expecting to obtain larger effect sizes overall. However, in contrast to our prediction, the long‐term clogging experiments had smaller effect sizes on ant patrolling (Table S2). For example, the short‐term clogging experiment had a 13% greater effect size in reducing numbers of ants patrolling extrafloral nectaries, 155% greater effect size increasing the numbers of ants inside flowers, and 132% greater increase of ant occupancy of flowers than the long‐term experiment (Figure 3b; Tables 1 and 2). Hence, we can robustly conclude that clogging the glands of only one leaf for 1 day is not too local and short‐term a treatment. In fact, leaf‐day is the scale at which we detected an effect of clogging and our experimental evidence showed that ant foraging behaviour responds to reward availability over this spatio‐temporal scale. The non‐provision of a whole branch for 10 days is a rather unnatural setting for ants, or may resemble a low‐rewarding plant (Lemus Domínguez, 2014).

Our results suggest that in *T. velutina* EFN‐mediated ant distraction is a mutualist management strategy that acts at a local and short‐term scale. This makes adaptive sense because plant structures vary in their vulnerability to herbivores and sensitivity to both benefits and costs of ant guards over similarly local and short‐term scales (Bentley1977b,; Falcão et al., 2014; Villamil, 2017; Willmer & Stone, 1997). From the plant's perspective, protection needs changes at a very small spatial and temporal scale (Bentley1977b,; Falcão et al., 2014; Villamil, 2017; Willmer & Stone, 1997) because in *T. velutina*, buds, flowers and fruits indeed occur in close proximity on the same shoot, and develop from bud to young fruit in only 3 days. Flowers are suggested to be the most vulnerable structure due to their soft and exposed water‐rich tissues, while buds and fruits are protected by the sepals or the exocarp respectively. Previous work has shown that EFN secretion in *T. velutina*is greatest at the flower stage, with glands in the associated leaf secreting 10 times more sugar than glands associated with fruit, and 40% more sugar than glands associated with buds (Villamil, 2017). This pattern of investment is compatible with both ODT (McKey, 1979; Ochoa‐López, Rebollo, Barton, Fornoni, & Boege, 2018; Ochoa‐López, Villamil, Zedillo‐Avelleyra, & Boege, 2015; Rhoades, 1979; Stamp, 2003) and the Distraction Hypothesis (for reduction of negative ant‐pollinator interactions).

From the ant's perspective, adjustment of foraging patterns at a local scale could maximise net sugar gain (Schilman & Roces, 2006). The rapid transition from bud to fruit in *T. velutina*means that secretion by individual glands can vary substantially over consecutive days since EFN secretion varies greatly throughout this transition (Villamil, 2017). Consequently, for the ants, missing the extrafloral nectaries of leaves associated with flowers means missing a bountiful reward.

### 4.5 Implications for ant and pollinator foraging strategies

We suggest that ants associated with *T. velutina* learn the location of highly rewarding EFN glands by monitoring variation in rewards within a single day, rather than relying on cues from previous days—a pattern compatible with demonstrated ability of ants to learn spatial and temporal scales of food rewards (Jackson & Morgan, 1993; Jackson & Ratnieks, 2006; Robinson, Jackson, Holcombe, & Ratnieks, 2005). There is also evidence that at least some pollinating insects can respond to similarly local variation in ant activity. Bees in other systems are known to use ant scents to discriminate and avoid heavily patrolled flowers, preventing harassment (Cembrowski, Tan, Thomson, & Frederickson, 2014) and we suggest that bees visiting *T. velutina*may also use olfactory cues to reduce their visitation of ant‐occupied flowers.

The local foraging decisions we propose and the effects of within‐plant variation in EFN availability on ants and pollinators should be seen as occurring against a backdrop of significant between‐plant variation in EFN rewards. Differences between individual plants, and not between branches or flowers, explained a large part of the variance in both numbers of ants and pollinators at both experimental scales (Table 1, Table S1). It is possible that plant‐level variation in nectar availability underlies the positive correlation between numbers of patrolling ants and pollinator visitation observed in the long‐term clogging treatment (Table S2), with each mutualist guild independently selecting more rewarding plants. Plant‐level variation in EFN rewards could have many causes, including phenotypic plasticity (Ochoa‐López et al., 2018), genetic variation in floral (Ramos‐Castro, 2013) and extrafloral nectar (Ochoa‐López, 2013; Ochoa‐López et al., 2018, 2015), and other variables such as plant age (Ochoa‐López et al., 2018; Villamil et al., 2013), size, floral display (Ramos‐Castro, 2013), proximity to a nest or hive (Cuautle & Rico‐Gray, 2003; Cuautle et al., 2005), plant vigour or soil fertility (Dattilo, Rico‐Gray, Rodrigues, & Izzo, 2013; Yamawo, Hada, & Suzuki, 2012).

## 5 CONCLUSIONS

Our findings on flower occupancy by ants at a leaf‐day scale support the Distraction Hypothesis suggesting extrafloral nectar secretion during anthesis can bribe ants away from flowers and significantly reduced ant occupancy of flowers. However, clogging EFN secretion did not result in a significant increase in ant abundance within flowers. This suggests that distraction via EFN secretion is neither the only nor the strongest mechanism in mutualist management by *T. velutina*. Further research is required to understand why ants rarely visit the flowers of *T. velutina*, and which mechanisms may be keeping ants outside these accessible, nectar producing flowers. Perhaps other mechanisms such as floral ant repellents (Ballantyne & Willmer, 2012; Willmer et al., 2009) reinforce ant exclusion. Differences in chemical composition or sugar concentration between floral and extrafloral nectars may also underlie observed ant foraging preferences. Further studies on a range of ant‐plants are required to assess the wider significance of EFN‐mediated ant distraction in amelioration of ant‐pollinator conflict.

## AUTHORS' CONTRIBUTIONS

N.V. conceived the ideas; all co‐authors contributed to the experimental design; N.V. carried out fieldwork experiments, and collected and analysed the data; N.V. led the writing of the manuscript, with critical inputs from K.B. and G.N.S.

## Data Availability

Data available from the Dryad Digital Repository: https://doi.org/10.5061/dryad.59f10v7 (Villamil, Boege, & Stone, 2019).
