# Supplementary material for: Testing the Distraction Hypothesis: Do extrafloral nectaries reduce ant‐pollinator conflict?
Source: J Ecol. 2019 Mar 6;107(3):1377–91. doi: 10.1111/1365-2745.13135 (PMC6559321; doi:10.1111/1365-2745.13135)
Supplement: Supplementary file 1 [file JEC-107-1377-s001.docx]

SUPPLEMENTARY MATERIAL 1

Long-term branch-scale reduction of EFN secretion

(10 leaves, 10 days)

# FIELDWORK METHODS

Based on the results from the short-term experiment, we conducted a follow-up experiment in which treatment duration and spatial scale were both increased by a factor of 10, using paired branches and focusing on one flower on each control or clogged branch, rather than paired flowers on the same branch (Fig. 1). In each of three sites, we selected 15 *T. velutina* plants bearing at least four apical flowering branches, each with ten leaves and reproductive structures (*n* = 45 plants). Two branches per plant were marked as control and two as clogged treatments using different string colours. We chose two branches to increase the probability of having one pair of focal flowers with each of the experimental treatments per plant per day. This experiment was conducted over 10 days during September 2015 for a total of 80 pairs of control and clogged flowers (n = 160 flowers). The extrafloral nectaries of all ten leaves on the clogged treatment branches were clogged as described in the methods for the short-term experiment, and the treatment was maintained for ten days. Glands were checked each day, and the paint was replenished if required, especially after heavy rains overnight. If new leaves emerged on a branch during the experiment, paint droplets were applied on or above their extrafloral nectaries according to their respective treatment (Fig. 1). Every morning all plants were checked and those bearing at least one pair of flowers with control or clogged treatment were observed following the same observational protocol as for the short-term experiment. Simultaneous observations were performed at each of three sites by different observers.

# STATISTICAL ANALYSES

Five mixed effects models with the same structure as those used to analyse the results from the short-term clogging experiment were fitted to analyse the long-term experiment data. Because all of these models had the same random effects structure, unless otherwise specified, we detail the random effects first and then describe the fixed effects for each model. In the long-term experiment, branch identity was fitted as a random effect to account for repeated hourly observations (Table S1). Because this experiment had a paired experimental design we fitted branch pair identity as a random effect to control for between-pair variation in floral and extrafloral investment. We also included an observation-level random effect where each data point received a unique level of a random effect to control for overdispersion. We fitted the following models, and have structured our results following this order:

(S.i) To test the effect of nectary clogging on the number of ants we fitted a Poisson mixed effects model using number of ants as the response variable. Ant location (at extrafloral nectaries or in flowers), treatment, and the interaction between these two factors were fitted as fixed effects. Tukey *post-hoc* tests were conducted in this model to find differences between the number of ants at extrafloral nectaries or flowers under control or clogged gland conditions.

(S.ii) To test whether preventing EFN secretion by clogging the glands increased the probability of a flower being occupied by ants, we fitted a binomial mixed effect model. We used the presence or absence of ants in a flower as a response variable, and fitted treatment as a fixed effect. The random effects remained the same, except for the observation-level random effect which we omitted.

(S.iii) To test the effect of clogging EFN secretion on pollinator visitation we fitted a Poisson mixed model using the number of pollinators as the response variable and treatment as the only fixed effect.

(S.iv) To test the whether the total number ants, regardless of their location, had an effect on pollinator visitation we fitted a Poisson mixed model using number of pollinators as the response variable. As fixed effects we fitted the total number of ants, and treatment to test whether treatment affected pollinator visitation in a way that was unlinked to the number of ants.

(S.v) To test if the location (inside flowers or at extrafloral nectaries) and number of ants affect pollinator visitation, we fitted a Poisson mixed model. The number of pollinators was fitted as the response variable, whilst treatment, number of ants in flowers, and number of ants at extrafloral nectaries were fitted as fixed effects.

Cohen *d* effect sizes for all models were calculated using the LRT statistics from each model. To test whether increasing the duration and scale of the clogging treatment by a factor of 10 had a larger effect on the number of ants and pollinators, we estimated the ratio of change in the effect size between the short- and long-term experiment for each type of visitor (Table S2).

# RESULTS AND DISCUSSION

## Reduction of EFN secretion

(S.i) EFN treatment (clogged *versus* control) and ant location both had significant effects on the number of ants, but with no significant interaction between these factors (Fig. S1b; Table S1). There were significant differences between the numbers of ants patrolling the nectaries and those inside flowers over both EFN treatments (Table S1). Almost seven times more ants were found patrolling extrafloral nectaries (1.53 ± 0.087) than were found inside the flowers (0.22 ± 0.034) (Fig. S1b; Table S1), regardless of EFN treatment (control: *Z* = -14.04, *P* < 0.001; clogged: *Z* = -11.41, *P* < 0.001). In contrast to the results from the short-term experiment, eliminating EFN secretion had a negative effect on the number of ants at both locations (Fig. S1b; Table 2). Clogging significantly reduced the number of ants at extrafloral nectaries (*Z* = -3.043, *P* = 0.011; Table 2), but had no significant effect on the number of ants in flowers (*Z* = -0.477, *P* = 0.96; Table 2) (Fig. S1b).

(S.ii) The percentage of flowers occupied by ants was 11% in both treatments, hence eliminating EFN secretion from all leaves did not increase ant visitation to flowers (Table S1).

(S.iii) Eliminating EFN secretion had no significant effect on the number of pollinators (Fig. S1b; Table S1).

(S.iv) The number of pollinators was significantly and positively correlated with the total number of ants found patrolling either the extrafloral nectaries or inside the flowers, regardless of the treatment (Tables S1, S2).

(S.v) Furthermore, when ant abundance was partitioned by their location on the plants between extrafloral nectaries and flowers, the number of ants patrolling extrafloral nectaries was significantly positively correlated with the number of pollinators (Table S1), whilst the number of ants inside a flower was significantly negatively correlated with the number of pollinators (Table S2). These results contrast strongly with patterns at the single flower scale.

In models (S.i)-(S.iii) used to analyse the long-term experiment data, differences between plants (captured by the plant random effect) explained more of the variation in the numbers of ants or pollinators than differences between branches (captured by the branch random effect) or random variation between observations (captured by the OLRE random effect) (Table S1). In models (S.iv, S.v) random variation between observations explained a larger proportion of the variation in the numbers of ants or pollinators, followed by plant identity (Table S1). Branch identity was the random effect that consistently explained least variation in the numbers of ants or pollinators (Table S1).

## Effect sizes

Increasing the duration and spatial scale of the clogging treatment by a factor of 10 did not result in larger overall effect sizes as we had predicted (Table S2). In fact, the direction of some effects was reversed when the scale was increased. For example, the impact of preventing EFN secretion on the number of ants inside flowers changed from positive at a short-term, local scale to negative in the long-term, branch-scale experiment (Table S2). The effect size of total ant numbers on pollinator activity changed from negative to positive when we increased the scale of the treatment, as expected. The same pattern was observed for the effect size of ants patrolling extrafloral nectaries on pollinator activity. However, the effect size of ants inside flowers on pollinator activity was reduced when we increased the scale of the treatment.

## Comparing effect sizes and spatio-temporal scales

Here we provide further discussion regarding other comparisons between the short and long-term clogging experiments that are beyond the scope of Distraction Hypothesis.

The effect of the total number of ants and the number of ants at EFN on pollinators was three times greater in the long-term scale experiment, as expected. Clogging extrafloral nectaries from a whole branch caused a 13 % greater decrease in the number of ants patrolling the EFN glands than clogging EFN glands from only one leaf. Since most ants are found patrolling EFN glands, this decreased the total ant numbers, and such reduction in ant patrolling may be driving the increase in pollinator visitation. However, the negative effect of ants inside flowers on pollinator visitation was reduced in the long-term experiment, supporting the idea that the Distraction Hypothesis operates at a leaf, daily scale.

The long-term experiment showed that numbers of ants inside flowers have a significant negative effect on the number of pollinators, as predicted by the Distraction hypothesis (Tables S1, S2). But the same experiment revealed two positive effects that are not predicted by the Distraction Hypothesis: between the total number of ants and pollinators, and between the number of ants patrolling extrafloral nectaries and pollinators (Tables S1-S2).

The positive effect of the number of ants on pollinator visitation may well represent the outcome of reward-based foraging decisions at the plant scale by both mutualists, leading to selection of more rewarding plants. Having all extrafloral nectaries within a branch clogged may resemble a low-rewarding plant, as explained in the main text. The positive relationship between ants and pollinators at a plant-scale (Table S2), may imply that plants of *T. velutina* able to recruit large numbers of ants do not experience a trade-off in pollinator visitation. Further experiments are required to test whether the ecological costs of ant patrolling on pollinator visitation differ between plants with high or low reward secretion.


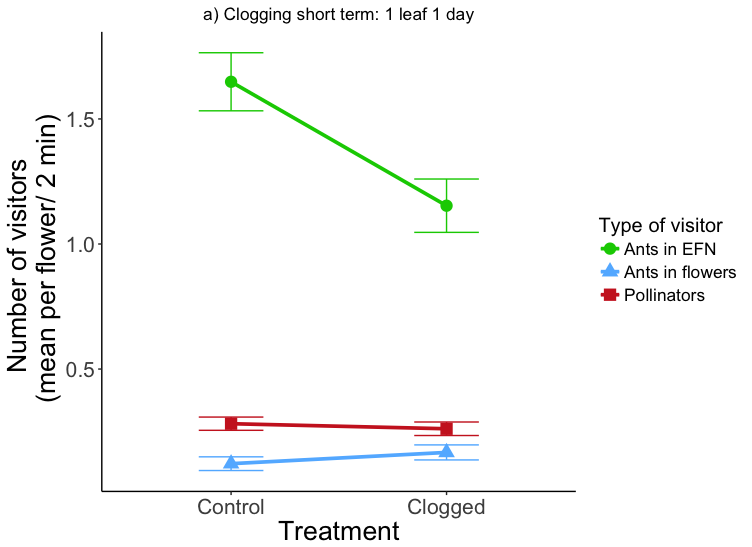

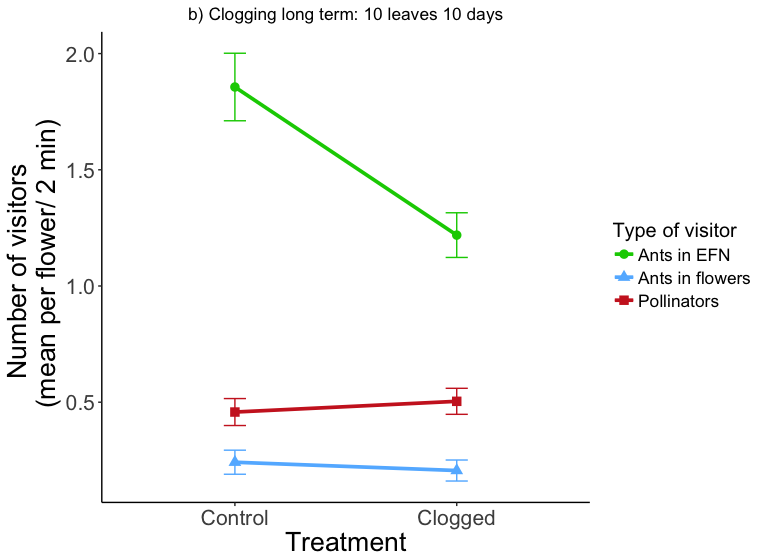


**Figure S1.** Effect of clogging the extrafloral nectaries on the number of visitors recorded in hourly surveys during 2 min of observation per flower (mean ± se) a) for the short-term experiment where one leaf was clogged for one day; and b) for the long-term experiment where ten leaves within a branch were clogged for ten days. Circles represent ants at extrafloral nectaries; triangles represent ant in flowers, and squares represent pollinators. Plot a) is also reported in the main text but has been repeated here to facilitate comparison with b).

**
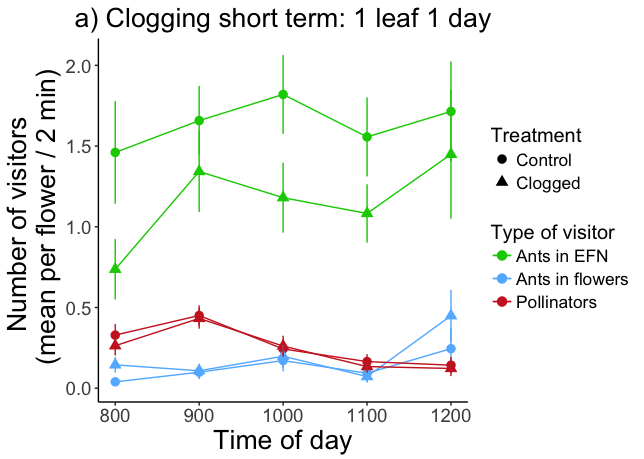
**
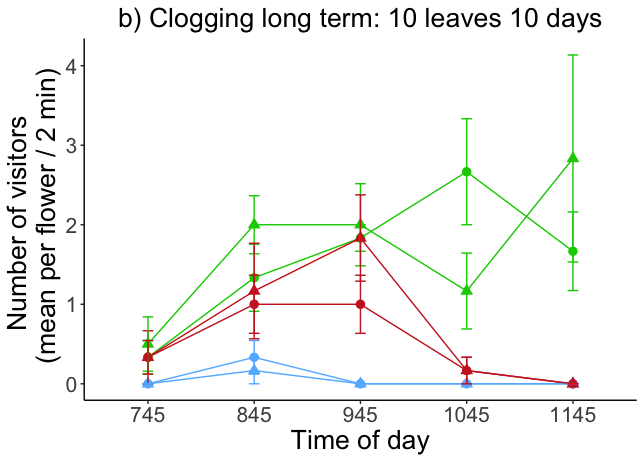
**Figure S2.** Hourly observations showing the effect of clogging of extrafloral nectaries on the number of visitors (mean ±se) a) for the short-term experiment in which one leaf was clogged for one day, and b) for the long-term experiment in which ten leaves within a branch were clogged for ten days.

**Table S1.** Likelihood ratio test results for statistical models used to test different response variables in the experiments. The values highlighted in bold are statistically significant (*P* > 0.05).

| **Experiment** | **Model** | **Response** | **Fixed effects** | **df** | **LRT** | ***P*-value** |  | **Random effects** | **Variance** | **SD** |
| --- | --- | --- | --- | --- | --- | --- | --- | --- | --- | --- |
| Long-term  Clogging  experiment |  | Number of ants | Clogging | 1 | 7.28 | **0.0069** | ** | Branch  Plant  OLRE | 0.20  0.30  0.60 | 0.45  0.55  0.77 |
|  | S.i) |  | Ant location  (EFN or flowers) | 1 | 395.5 | **2.2^-16^** | *** |  |  |  |
|  |  |  | Clogging × Ant location | 1 | 1.79 | 0.18 |  |  |  |  |
|  |  | Proportion of flowers occupied by ants | Clogging | 1 | 16.49^-5^ | 0.98 |  | Branch  Plant | 9.87^-10^  1.07 | 3.14^-10^  1.03 |
|  | S.ii) |  |  |  |  |  |  |  |  |  |
|  | S.iii) | Number of pollinators | Clogging | 1 | 0.63 | 0.42 |  | Branch  Plant  OLRE | 1.04^-9^  0.39  0.54 | 3.23^-05^  0.62  0.73 |
|  |  | Number of pollinators | Clogging | 1 | 1.43 | 0.23 |  | Branch  Plant  OLRE | 0  0.39  0.48 | 0  0.63  0.69 |
|  | S.iv) |  | Total number of ants | 1 | 5.17 | 0.02 | * |  |  |  |
|  |  | Number of pollinators | Clogging | 1 | 2.00 | 0.15 |  | Branch  Plant  OLRE | 0  0.38  0.41 | 0  0.62  0.64 |
|  | S.v) |  | Ants in EFN | 1 | 10.85 | **0.0009** | *** |  |  |  |
|  |  |  | Ants in flowers | 1 | 3.78 | **0.05** | * |  |  |  |

**Table S2.** Comparison of the effect sizes of the short and long-term clogging experiments on the number of visits per visitor type.

| **Model** | **Short term experiment:**  Clog 1 day 1 leaf | | | **Long term experiment:**  Clog 10 days 10 leaves | | | **Effect size difference ratio** |
| --- | --- | --- | --- | --- | --- | --- | --- |
|  | **Fixed effect** | ***d*** | **Effect size** | **Fixed effect** | ***d*** | **Effect size** |  |
| i) | Ants in EFN | -0.2865 | Small | Ants in EFN | -0.2489 | Small | -13% |
| i) | Ants in flowers | +0.070 | Small | Ants in flowers | -0.0387 | ns | -155% |
| ii) | Flowers occupied by ants | +0.146 | Small | Flowers occupied by ants | +0.011 | ns | -132% |
| iii) | Pollinators | +0.0503 | ns | Pollinators | +0.0698 | ns | +38% |
| iv) | Clogging | -0.0488 | ns | Clogging | +0.1053 | ns | +315% |
| iv) | Total ants | -0.0915 | ns | Total ants | +0.2065 | Small | +325% |
| v) | Clogging | -0.0676 | ns | Clogging | +0.1245 | ns | +284% |
| v) | Ants in EFN | -0.1314 | ns | Ants in EFN | +0.3054 | Medium | +332% |
| v) | Ants in flowers | -0.0893 | ns | Ants in flowers | -0.1555 | ns | -74% |

SUPPLEMENTARY MATERIAL 2

Ant species-specific responses to clogging and effects on pollinators

# STATISTICAL ANALYSES

## Effects of clogging on ant species

(S.vi) To investigate whether ant species differed in their response to clogging we fitted a Poisson mixed model using the number of ants inside the flower as the response variable, and treatment as a fixed effect. As random effects we fitted flower identity, pair identity, an observation-level random effect to deal with overdispersion, ant species, and the interaction between treatment and ant species. This model allowed us to estimate the amount of variance in the number of ants inside flowers explained by ant species, and estimate the effect of clogging on the abundance of each ant species inside flowers.

Although the ant species and the interaction between clogging and ant species explained a negligible amount of variation in the abundance of ants inside flowers, we extracted the random effect estimates for our “species” and “species x clogging” random effects. For the random effect “ant species” in model (S.vi) we extracted the estimates for every level within this random effect and its standard error (Fig. S3a).

## Effects of ant species on pollinators

To investigate whether different ant species patrolling the plants or inside the flowers differed in their effect on pollinator visitation, we fitted multiple membership mixed models using MCMCglmm (Hadfield, 2010). In these models, we fitted the abundance of ants from each species as covariates and treated the regression coefficients as random. This allowed us to estimate the effects that different species of ant have on the number of pollinators.

These statistical tools allow us to account for plants occupied by multiple ant species, and capture the variation in ant abundance within a given species. This model also prevents us from making *a priori* assumptions of ant attributes that may make certain species more prone to responding to clogging or ant attributes that may have an effect on pollinator visitation. Such *a priori* assumptions may be flawed due to the lack of previous studies testing this. Instead, these models allow us to detect the magnitude and direction (positive or negative) of the effect each ant species has on pollinator visitation. Using this approach allows us to generate robust evidence-based assumptions of ant species attributes that may enhance or hinder pollinator visitation.

(S. vii) To investigate if the number of ants of different species inside the flowers affected pollinator visitation, we fitted an ordinal multi-membership mixed model. Although the data are count data we chose to treat the number of pollinator visits as ordinal because the number of visits fell into a few categories (0, 1, 2, or 3) and the mean and variance of the counts were equal suggesting that the data would be underdispersed with respect to the Poisson if there was real heterogeneity in pollinator visitation rates. The number of pollinators was fitted as the response variable, whilst treatment, number of ants in flowers, and number of ants at extrafloral nectaries were fitted as fixed effects. As random effects we fitted flower, pair identity, and an observation-level random effect to account for overdispersion. The regression coefficients of the number of ants inside flowers from each species were treated as random additive effects. Fitting these additive random effects allowed us to estimate the effects that different ant species inside flowers had on pollinator visitation.

(S.viii) To investigate whether the number of ants of different species patrolling the extrafloral nectaries differed in their effect on pollinator visitation, we fitted an ordinal multi-membership mixed model. The number of pollinators was fitted as the response variable, whilst treatment, number of ants in flowers, and number of ants at extrafloral nectaries were fitted as fixed effects. As random effects we fitted flower and pair identity, and an observation-level random effect to account for overdispersion. We also fitted the abundance of ants from each species at the extrafloral nectaries as random additive effects which allowed us to estimate the amount of variance in the number of pollinators explained by ant species, and estimate the effects of patrolling by each ant species on pollinator visitation.

## RESULTS AND DISCUSSION

**Effects of clogging on ant species**

(S.vi) We found no significant effect of clogging on the number of ants inside flowers (Table S3), which confirmed the results from model (i) in the short-term experiment. The interaction between clogging and ant species explained no variation in the number of ants inside flowers. Most of the variation was explained by the differences between observations (OLRE: 97 %), followed by pair identity (2.3 %), and finally ant species explaining only 0.21 % of the variation. The estimates of the between-species effects are low and imprecise (Fig. S3a). The imprecision is for two reasons; more common species are sampled more often, whilst rare species are seldom sampled, hence their confidence intervals are larger and estimates less precise. Secondly, because overall ants inside the flowers is a rare phenomenon (Fig. S1). The qualitative patterns obtained from the “ants species x clogging” effects helped us detect ant species with the greatest differences between control and clogged (Fig. S3a) which would be worth exploring in a controlled experimental way. These species are: *Brachymyrmex, Paratrechina longicornis, C. planatus*, and *C. mucronatus*.

**Effects of ant species on pollinators**

(S.vii) The effects of different ant species at extrafloral nectaries on pollinator visitation are precisely estimated by our models, (see SE in Fig. S3b). All effects overlap with zero, revealing a negligible effect of ant species at extrafloral nectaries on pollinator visitation (Table S4). We could detect qualitative patterns in this data: *Dorymyrmex bicolor* ants have a on average, negative effect on pollinator visitation, whilst *Camponotus mucronatus* and *Paratrechina longicornis* have, on average, a positive effect on pollinator visitation (Fig. S3b).

(S.viii) The effects that different ant species inside flowers have on pollinator visitation are imprecisely estimated by our models, as indicated by the large SE values associated with these estimates (Fig. S3c). Qualitative patterns in this data suggest that when inside flowers, *Dorymyrmex bicolor* ants may have a negative effect on pollinators. Such patterns also help us detect ant species whose positive or negative effects on pollinators would be worth exploring in further experimental work. Furthermore, these results are consistent with experimental findings demonstrating that *Dorymyrmex bicolor* ants placed inside flowers of *T. velutina* induce alert behaviours in pollinators, reduce visit duration, increasing handling time per flower and reducing foraging efficiency (Villamil et al., 2018).

a)

b)
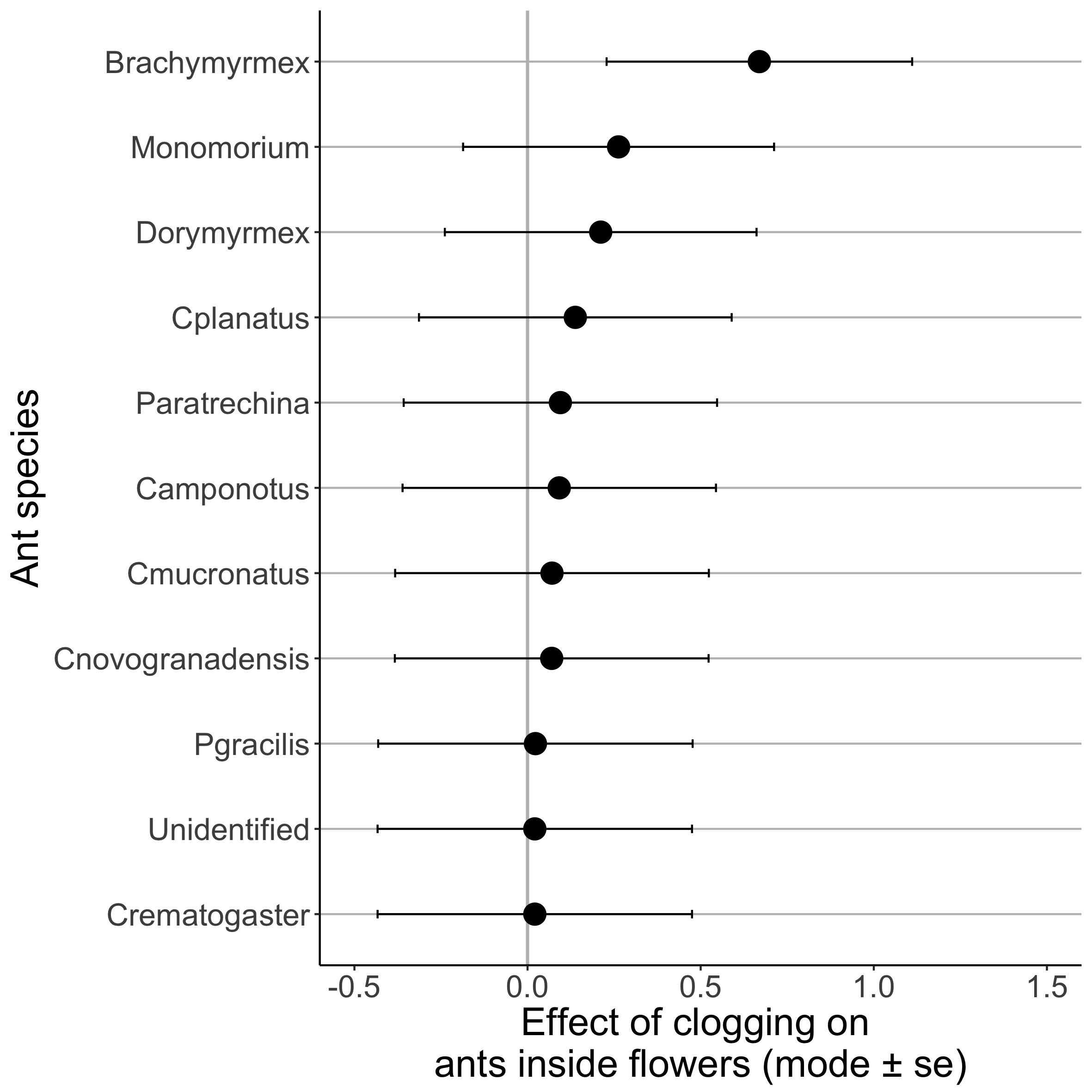


c)
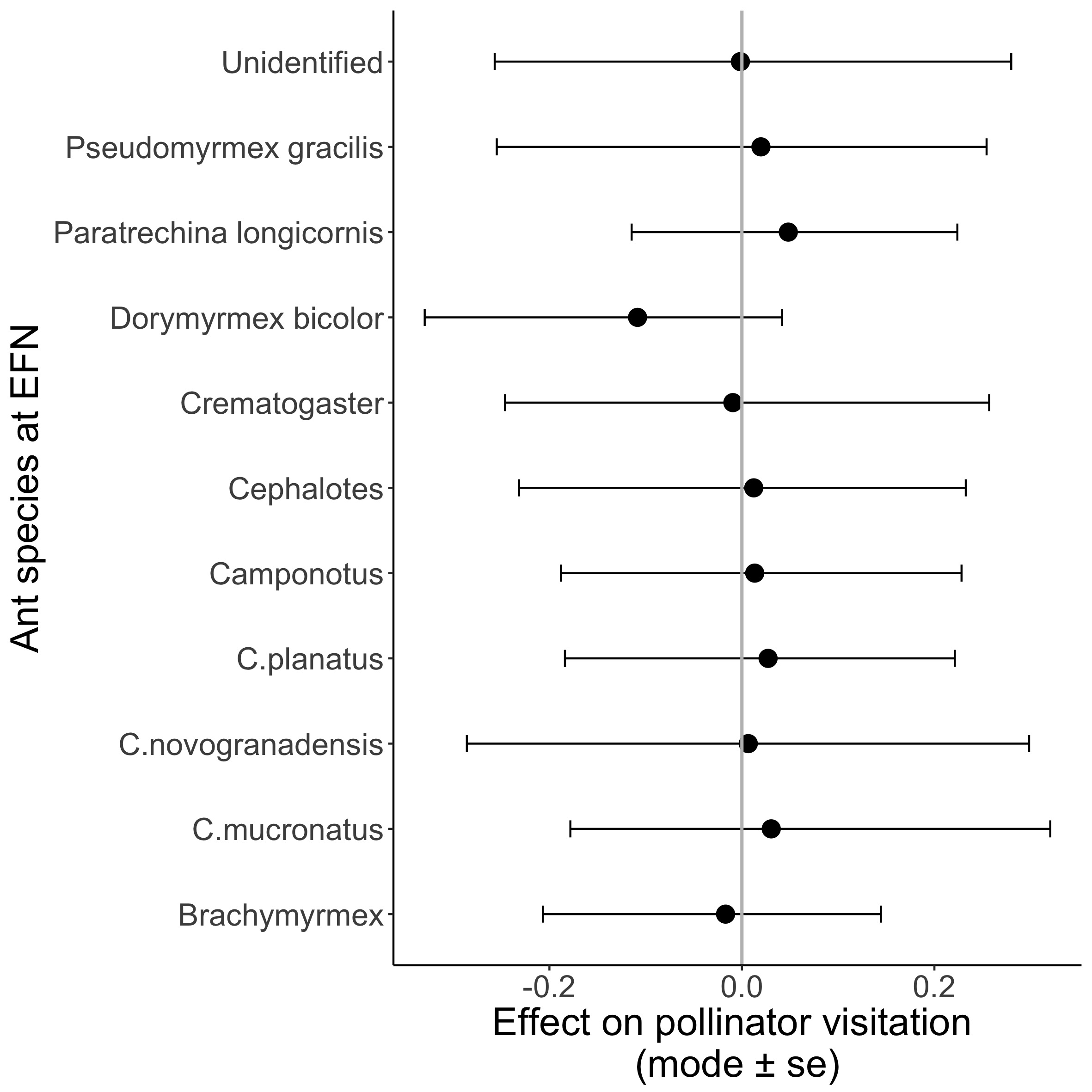


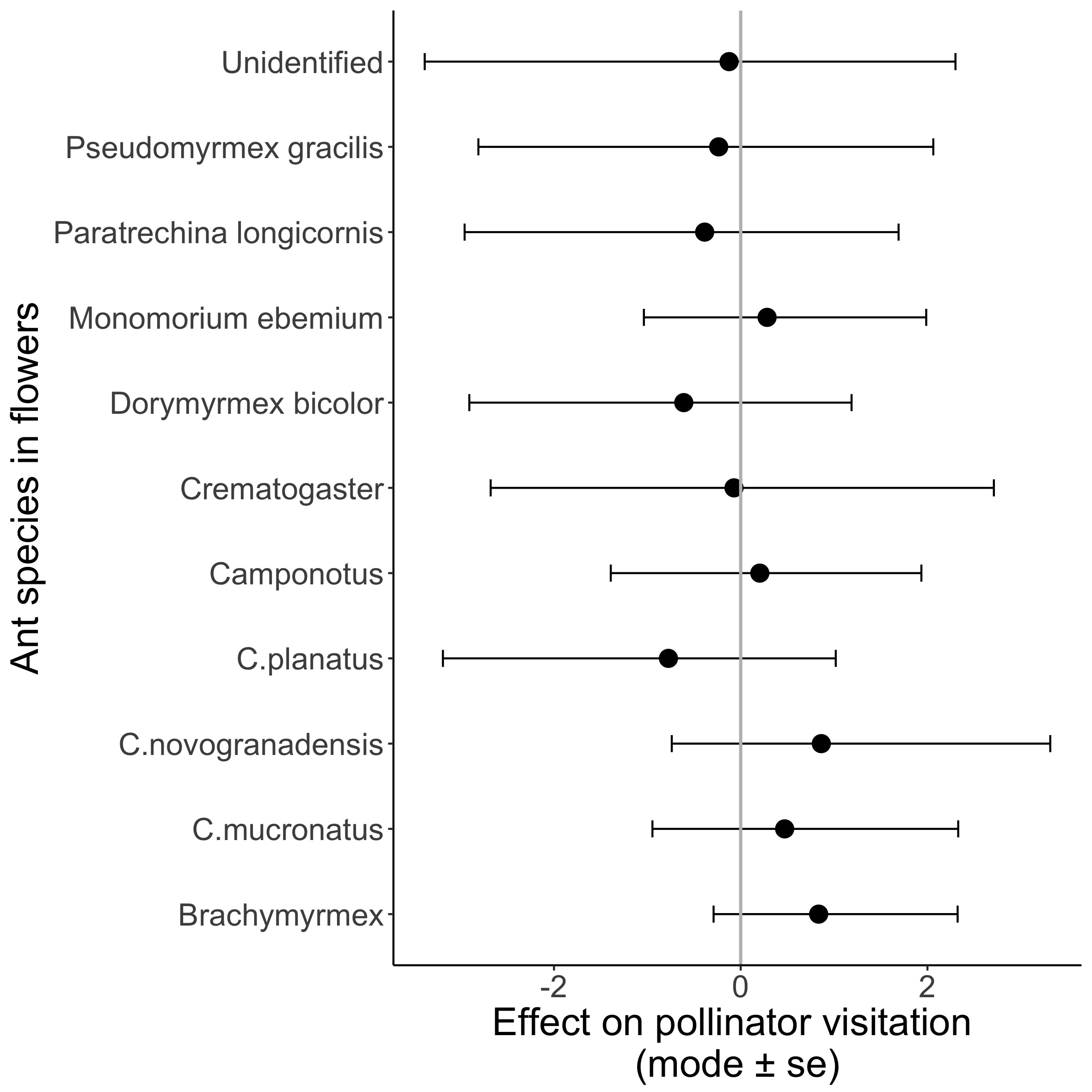


**Figure S3.** a) Effect of clogging the extrafloral nectaries on different ant species inside flowers and effects of different ant species on pollinators, and their precision. Effect of ant species (b) patrolling extrafloral nectaries, or (c) inside the flowers on pollinator visitation (mode ± SE).

**Table S3.** Mean effect of each ant species patrolling at EFN glands on pollinator visitation using a multiple membership mixed model.

| **Model** | **Response** | **Fixed effects** | **Estimate** | **LRT** | ***P*-value** |  | **Random effects** | **Variance** | **SD** |
| --- | --- | --- | --- | --- | --- | --- | --- | --- | --- |
| S.vi) | Ants inside flowers | Clogging | 0.55 | 0.61 | 0.43 |  | Species  Species x Clogging  Flower id  Pair id  OLRE | 0.20  6.14^-10^  0  2.20  90.41 | 0.45  2.47^-05^  0  1.48  9.50 |
|  |  |  |  |  |  |  |  |  |  |

**Table S4.** Mean effect of each ant species patrolling at EFN glands on pollinator visitation using a multiple membership mixed model.

| **Model** | **Response** | **Fixed effect** | **Post mean** | **95% CI lwr** | **95% CI upr** | **Random effects** | **Variance** | **95% CI lwr** | **95% CI upr** |
| --- | --- | --- | --- | --- | --- | --- | --- | --- | --- |
| s.vii | Number of pollinators | Clogging | -0.1803 | -0.4725 | 0.0959 | Flower id | 0.02372 | 3.83^-09^ | 0.08859 |
|  |  | Ants at EFN | -0.1208 | -0.2717 | 0.0261 | Pair id | 1.234 | 0.6468 | 1.935 |
|  |  | Ants in flowers | -0.1377 | -0.5213 | 0.2409 | Ants at EFN | 0.02008 | 2.21^-11^ | 0.07075 |
|  |  |  |  |  |  |  |  |  |  |
| s.viii | Number of pollinators | Clogging | -0.1707 | -0.4460 | 0.1155 | Flower id | 0.02506 | 5.37^12^ | 0.0969 |
|  |  | Ants at EFN | -0.1479 | -0.2601 | -0.0402 | Pair id | 1.326 | 0.7137 | 2.104 |
|  |  | Ants in flowers | -0.7854 | -2.2555 | 0.3802 | Ants in flowers | 2.364 | 7.284^-07^ | 9.139 |
